# Ultrasound Imaging for a Rare Cause of Sciatica: A Schwannoma of the Sciatic Nerve

**DOI:** 10.7759/cureus.8214

**Published:** 2020-05-20

**Authors:** Wei-Ting Wu, Ke-Vin Chang, Yu-Chun Hsu, Yi-Chiang Yang, Po-Cheng Hsu

**Affiliations:** 1 Physical Medicine and Rehabilitation, National Taiwan University Hospital, Bei-Hu Branch, Taipei, TWN; 2 Physical Medicine and Rehabilitation, Taipei Veterans General Hospital, Taipei, TWN

**Keywords:** hip, schwannomas, sciatic nerve, ultrasound

## Abstract

Sciatica is a common musculoskeletal complaint, but it is rarely attributed to peripheral nerve tumors. Until now, there is little literature reporting sciatica caused by a sciatic schwannoma at the proximal thigh. A 27-year-old male had left posterior proximal thigh pain for more than two years. Compression of the tender point caused numbness radiating to his low back, buttock and leg regions. Due to poor response to conservative treatments, he was referred for an ultrasound examination, which revealed a solid mass on the track of the sciatic nerve. The subsequent magnetic resonance imaging showed a well-defined tumor sized 2.3 × 1.8 × 2.3 cm beside the sciatic nerve, and a schwannoma was confirmed by postsurgical pathology. In conclusion, ultrasound is helpful in differentiating between the various causes of posterior thigh pain, which, in this case, facilitated detection of a sciatic nerve schwannoma and subsequent surgical removal.

## Introduction

Sciatica is a prevalent musculoskeletal complaint, presented with pain radiating from the back to the buttock, thigh and leg [1]. Some cases of sciatica are associated with pathology of the intervertebral discs, aggravated by increased intra-abdominal pressure after coughing or sneezing [2]. Other possible etiologies include degenerative lumbar-sacral radiculopathy, pyriformis syndrome and peripheral neuropathy.

Sciatic nerve neoplasm is an uncommon cause of sciatica, which cannot be easily diagnosed by physical examinations. The rapid development of high-resolution ultrasound allows clear depiction of peripheral nerve pathology. In case of a neurogenic tumor, early diagnosis with prompt intervention leads to speedy recovery and prevention of subsequent motor and sensory deficits. In this regard, we would like to report a case with chronic sciatica caused by a schwannoma and the utility of ultrasound in diagnosis.

## Case presentation

A 27-year-old male, without any family history of the cancer events, suffered from left posterior thigh pain for more than two years. The pain had an insidious onset and became more intense gradually. He denied any pre-existing neurological diseases and trauma at the painful region. He had visited several doctors where possible diagnoses like intervertebral disc herniation, lumbar-sacral radiculopathy, myofascial pain syndrome and fibromyalgia were given. Various kinds of therapies had been applied, such as local injection, lumbar traction, heat thermal modalities, manual exercise and acupuncture. At his worst moment, oral Ultracet (tramadol 37.5 mg/acetaminophen 325 mg) and pregabalin 75 mg were needed for pain control. In recent few months, the pain had made it difficult for him to sit on the chair. He was then referred to our clinic for better management.

**Figure 1:**
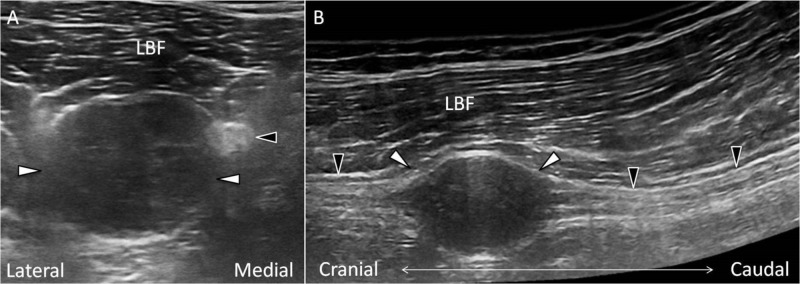
Ultrasonography of the sciatic schwannoma A tumor was noted beside the sciatic nerve with posterior enhancement in its short axis view (A). In the panoramic view, the tumor was noted below the long head of biceps femoris. The sciatic nerve bundles were seen at the cranial and caudal ends of the tumor (B). Black arrowheads: sciatic nerve; white arrowheads: schwannoma. LBF: long head of biceps femoris.

During physical examination, neither tenderness nor numbness was noted at the paraspinal and gluteal areas. No radiating pain was elicited by lumbar flexion, extension, rotation and lateral bending. The straight leg raising test yielded a negative finding. The Tinel sign was positive when compressing his left posterior mid-thigh, and the numbness and tingling sensation were radiated to the lumbar, gluteal and leg regions. The muscle power and sensation (light touch and pinprick) were intact at both lower limbs. We performed an ultrasound examination of his left posterior thigh. A solid mass sized 2 cm × 1.7 cm × 2.2 cm with clear posterior enhancement was noted on the track of the sciatic nerve (Figure 1A). There was a well-defined border between the mass and surrounding tissues (Figure 1B).

**Figure 2:**
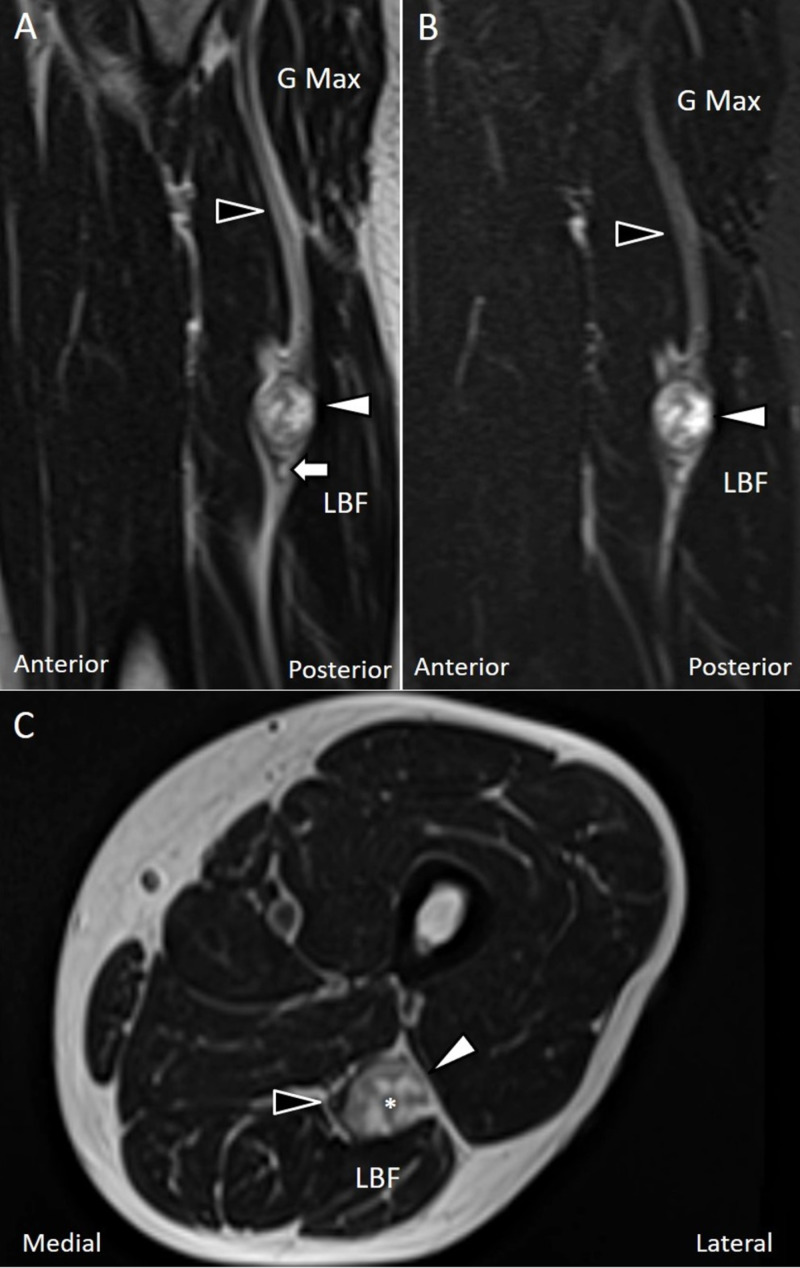
MRI of the sciatic schwannoma A well-defined tumor of dimensions 2.3 × 1.8 × 2.3 cm was noted in the posterior compartment of the left thigh under the T2-weighted non-contrast (A) and T1-weighted contrast-enhanced MRI (B). On the axial plane, the tumor was located beside the left sciatic nerve (C). Black arrowheads: sciatic nerve; white arrowheads: schwannoma; white arrow: split-fat sign; asterisk: fascicular sign. G Max: gluteus maximum; LBF: long head of biceps femoris.

We did not visualize a significant increase in power Doppler signals inside or adjacent to the mass. The magnetic resonance imaging (MRI) with contrast of gadolinium was arranged, revealing a well-defined mass located in the posterior compartment of the left thigh. The mass, abutting the left sciatic nerve, revealed a pattern of heterogeneous hyperintensity on T2-weighted imaging with the rim of fat around it (Figure 2A). Following the administration of contrast media, multiple ring-like enhancement indicating intraneural fascicular bundles was observed within the mass (Figure 2B). The nerve fascicles from the sciatic nerve were deviated medially to the mass (Figure 2C).

Under the impression of a neurogenic tumor, he received neurolysis and the pathology confirmed it to be a schwannoma. After the excision, the patient did not have any neurological deficit and achieved a complete relief of the pain.

## Discussion

Peripheral nerve sheath tumors include benign lesions, or malignant tumors [3]. In benign peripheral nerve sheath tumors, schwannomas and neurofibromas should be distinguished. Schwannomas (also called neurilemoma), arising from the Schwann cells of the neural sheath, are the most common peripheral nerve tumors and frequently emerge from the main nerve trunk [4]. Schwannomas commonly occur in adults between 20 and 50 years of age without sex predilection. Most schwannomas are benign and present as fusiform- or oval-shaped encapsulated masses, causing compression and lateral displacement of the adjacent nerve [5]. In contrast, neurofibromas, another prevalent nerve sheath tumor associated with neurofibromatosis type 1, have a mixed cell origin including Schwann cells, fibroblasts, mast cells and axons [6,7]. Owing to the fact that the fibrous tissues are likely to located at center of the mass and myxoid substances are distributed peripherally, the target sign may be observed on MRI [8]. However, neurofibromas are mostly associated with neurofibromatosis and have a higher risk of malignant transformation. Unlike benign tumors, the malignant tumors have the characteristics of larger size, ill-defined margins, central necrosis and surrounding tissue infiltration [8]. Complete excision is the best treatment option for peripheral nerve sheath tumors. Compared with schwannomas, the outcomes after neurofibroma excision are not always satisfactory because of variable grades of tumor infiltration into the accompanying nerves.

The sciatic nerve receives fibers from the L4 to S3 nerve roots. It descends superficial to the adductor magnus muscle, between and below the medial side of the biceps femoris and lateral side of the semitendinosus and semimembranosus muscles [9]. Schwannomas from the sciatic nerve are rare and less likely to have malignant transformation. There is a low risk of recurrence after schwannoma excision [5]. If a patient presents with tenderness over the posterior thigh but is irresponsive to conservative management, physicians should be alert to possible malignancy involving in the sciatic nerve [10].

MRI and ultrasound are common imaging modalities for investigating peripheral nerve disorders. On T1-weighted MRI images, schwannomas are seen with rims of fat in their proximal and distal ends, known as the “split fat sign”. On T2-weighted images, the tumors have multiple hypointense foci interspersed in a hyperintense background, called the “fascicular sign” [8,11]. According to Jee et al., the fascicular sign is more commonly noticed in schwannomas than in neurofibromas [12]. Compared with MRI, ultrasound has the advantages of easy accessibility, portability and allowance of dynamic examination.

Under ultrasound imaging, schwannomas appear as well-defined, homogeneous, hypoechoic and fusiform or round masses. The sign of posterior enhancement is easily viewed because of the myxoid substance with high water content in the tumor [11]. Using the power Doppler ultrasound imaging, hyperemia may be occasionally seen in schwannomas but less frequently visualized in neurofibromas [13].

Some clinical pearls are worth sharing regarding ultrasound investigation for patients with chronic sciatica. First, the investigators should be aware of the fact that the sciatic nerve may be invisible underneath the acoustic shadow casted by the conjoint tendon of the biceps femoris and semitendinosus tendons [14]. The transducer can be swept to the medial or lateral aspect of the posterior thigh to avoid this potential blind spot. Second, entrapment of the posterior femoral cutaneous nerve can elicit symptoms like sciatica. The examiner can easily identify the nerve at the mid-thigh level superficial to the interval between the long head of the biceps femoris muscle and the semitendinosus muscle [15].

## Conclusions

The present case report demonstrated the utility of ultrasound in differentiating the various causes of posterior thigh pain. The physician should be aware of the possibility of schwannomas on the sciatic nerve in cases with recalcitrant sciatica.
